# Aluminum‐Doped Cesium Lead Bromide Perovskite Nanocrystals with Stable Blue Photoluminescence Used for Display Backlight

**DOI:** 10.1002/advs.201700335

**Published:** 2017-07-31

**Authors:** Ming Liu, Guohua Zhong, Yongming Yin, Jingsheng Miao, Ke Li, Chengqun Wang, Xiuru Xu, Clifton Shen, Hong Meng

**Affiliations:** ^1^ School of Advanced Materials Peking University Shenzhen Graduate School Shenzhen 518055 China; ^2^ Center for Photovoltaics and Solar Energy Shenzhen Institutes of Advanced Technology Chinese Academy of Sciences Shenzhen 518055 China

**Keywords:** anion‐exchange effect, blue photoluminescence, doping, perovskite nanocrystals, white‐light‐emitting diodes

## Abstract

Bright and stable blue emitters with narrow full‐width at half‐maxima are particularly desirable for applications in television displays and related technologies. Here, this study shows that doping aluminum (Al^3+^) ion into CsPbBr_3_ nanocrystals (NCs) using AlBr_3_ can afford lead‐halide perovskites NCs with stable blue photoluminescence. First, theoretical and experimental analyses reveal that the extended band gap and quantum confinement effect of elongated shape give rise to the desirable blueshifted emission. Second, the aluminum ion incorporation path is rationalized qualitatively by invoking fundamental considerations about binding relations in AlBr_3_ and its dimer. Finally, the absence of anion‐exchange effect is corroborated when green CsPbBr_3_ and blue Al:CsPbBr_3_ NCs are mixed. Combinations of the above two NCs with red‐emitting CdSe@ZnS NCs result in UV‐pumped white light‐emitting diodes (LED) with an National Television System Committee (NTSC) value of 116% and ITU‐R Recommendation B.T. 2020 (Rec. 2020) of 87%. The color coordinates of the white LED are optimized at (0.32, 0.34) in CIE 1931. The results suggest that low‐cost, earth‐abundant, solution‐processable Al‐doped perovskite NCs can be promising candidate materials for blue down‐conversion layer in backlit displays.

## 1 Introduction

Driven by the demands for lighting and display with both low power consumption and perfect visual effect, various colloidal nanocrystals (NCs) based white‐light‐emitting diodes (WLEDs) have been explored.^1^ Compared with traditional metal‐chalcogenide NCs, halide perovskites NCs possess unique characteristics of wide color tunability via postsynthesis anion exchange and good photochemical stability even without shell coating.^2^ In particular, all‐inorganic cesium lead halide (CsPbX_3_, *X* = Cl, Br, I) NCs deliver prominent optical‐gain signatures that combine the advantages of both quantum dots and halide perovskites: high color‐purity with narrow tunable photoluminescence (PL) emission, high quantum efficiency, and higher stability compared with their organic–inorganic hybrid counterparts.^3^ These features are ideal for achieving saturated colors and enriching the display or television color gamut, which make them an promising candidate for backlight applications.

Dialectically, the fast anion exchange reaction between different halide NCs has caused issues in broadening emission spectra and chromaticity drifts while using this great concept for color tuning.^4^ Recently, some examples have been demonstrated to resolve this problem. Green emitting bromide‐based and red emitting iodide‐based perovskite NCs were embedded in a polymer orsilica matrix.^5^ Yet most of these reported WLEDs are still based on a device structure utilizing a blue LED chip, on top of which perovskite NCs are coated. In fact, ultraviolet‐pumped WLEDs (UV‐WLEDs) are potential candidates, which can reduce blue light over exposure to an eye from current blue chip‐WLEDs. For UV‐WLEDs, blue emitting NCs with high efficiency are necessarily to complete blue component of the white light. However, blue emitting chloride‐based perovskite NCs usually suffered low brightness and the interparticle anion‐exchange issues as well. While the quantum confinement in bromide‐based nanoplatelets can shift the emission wavelength from green to blue, the formation of larger non‐quantum‐confined aggregates are unfavorable to generate stable white light.^6^ Even though other extreme measures, such as the use of X‐ray irradiation under vacuum to improve the stability, have implemented,^7^ the challenge remains. Development of novel perovskite NCs that not only preserve bright but also provide stable blue photoluminescence without strong anion exchange issue is the best way to proceed.

Intriguingly, halide perovskites enjoy great compositional flexibility. The electronic and optical properties of lead halide NCs can be also fine‐tuned by substitution of the cations. Mixed‐cation perovskites, such as Rb‐Cs polynary hybrid perovskite and Pb‐In binary metal perovskite, have already been synthesized as highly efficient photovoltaic materials with better stability and optimal bandgap.^8^ These results stimulate the research to exploit mixed‐cation halide perovskites light‐emitting materials with special optical characteristics. For light emitting application, doping of lead cation site maybe more effective in the band engineering than doping of mono‐valent cation site because the upper valence band is formed predominately by the halide p‐orbitals and the lower conduction band is formed by the overlap of the lead p‐orbitals. For example, cation substitutions of CsPbBr_3_ NCs with CH_3_NH_3_^+^ resulted in a peak emission shift from 510.2 to 525.4 nm, similar to that of CH_3_NH_3_PbBr_3_ (527 nm).^9^ For lead cation site, in both bulk and nanocrystalline forms, color‐tuning by partial lead substitution during the synthesis has been proven successful. Bi (III)‐doped organometal halide perovskites films exhibit edultra wide emission spectra in the near infrared region (850–1600 nm).^10^ Mn (II)‐doped lead chloride perovskite NCs possess dual‐color emission.^11^ So far, no demonstration of desirable blueshifted emission has been reported using doping strategy. This guided our motivation to study blue‐emissive perovskites NCs by the incorporation of other impurity (i.e., ions) into substituted CsPbBr_3_ nanocrystalline systems.

In order to provide stable green‐emission and avoid the anion exchange effect within different halide perovskite NCs for WLEDs, CsPbBr_3_ was selected as the host semiconductor material. Aluminum is one of the earth‐abundant elements belonging to IIIA group and classically used in the state‐of‐the‐art GaN‐based blue light emitting diodes. Most commonly, aluminum loses its three outermost electrons and becomes aluminum(III) ions (Al^3+^) to a stable oxidation state. In this study, we propose and investigate an alternative blue‐emissive material, Al (III)‐doped CsPbBr_3_ (Al:CsPbBr_3_) perovskite NCs, where some of Pb(II) atoms are substituted by Al(III). The synthetic approach for producing Al:CsPbBr_3_ NCs is based on a modified hot injection method, using AlBr_3_ as the precursor. The absence of anion exchange effect was corroborated when green‐emitting CsPbBr_3_ and blue‐emitting Al:CsPbBr_3_ NCs were mixed. Combinations of the green and blue all‐inorganic perovskite NCs with red‐emitting CdSe@ZnS NCs resulted in UV‐pumped WLEDs with an National Television System Committee (NTSC) value of 116% and ITU‐R Recommendation B.T. 2020 (Rec. 2020) of 87%.

## 2 Results and Discussion

**Figure 1:**
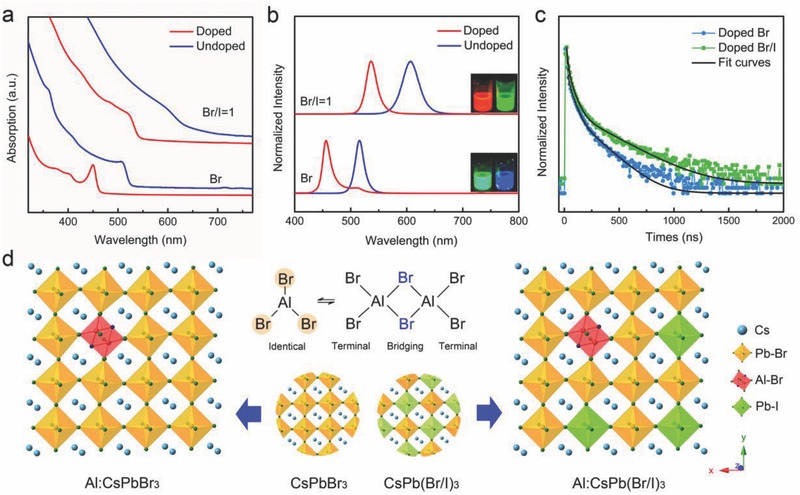
a) Absorption spectra of Al‐doped and undoped CsPbBr_3_ and CsPb(Br/I)_3_ nanocrystals. b) Photoluminescence of Al‐doped and undoped CsPbBr_3_ and CsPb(Br/I)_3_ nanocrystals. Insets are photographs of the sample under UV excitation. c) Time‐dependent Al luminescence intensity from Al‐doped CsPbBr_3_ and CsPb(Br/I)_3_ nanocrystals. d) Schematics showing the Al bound to host lattice constituents in cluster form.

The absorption and emission spectra of Al (III)‐doped and undoped CsPbBr_3_ NCs are shown in **Figure** **1**. The addition of aluminum atoms results in a blueshift of both the first exciton absorption and the emission peaks. The Al‐doping concentration determined from elemental analysis is ≈0.2% for Al:CsPbBr_3_ NCs. While the original CsPbBr_3_ NCs exhibited green emission at 515 nm, the resulting Al:CsPbBr_3_ NCs showed deep‐blue emission at 456 nm with a narrow full width at the half‐maximum of 16 nm. The possible sources of spectral shifts in such quantum‐confined particles may be associated with electronic doping by aluminum ions or related to size changes upon doping. The electronic structure and density of states of Al‐doped CsPbBr_3_ was calculated using VASP code (Figures S1–S3, Supporting Information). Al doping introduce a new level in the bandgap, which is contributed by the hybridization of the Al s‐orbitals, the Br p‐orbitals, and the Pb p‐orbitals. Al‐Pb binary metal perovskites demonstrate extended bandgap, indicating the experimental results are in good agreement with this theoretical calculation trend.

Generally, the synthesis of doped colloidal semiconductor NCs is really a tough work since the incorporation of ions in the lattice of small nanocrystals is usually kinetically unfavorable. However, the several reports showed accomplished doping examples via delicate kinetic control over surface morphology, nanocrystal shape, surfactants, or bond dissociation energy, indicating that the difficulties with doping could be not intrinsic.^12^ For lead halide perovskite system, the similar bond energy between dopant precursor and Pb‐X is considered to be one of the key factors that favors impurity incorporation.[[qv: 11a]]

In this work, AlBr_3_ was used as the precursor for introducing no other impurity than Al ion in CsPbBr_3_ NCs. The dimeric form of aluminum tribromide (Al_2_Br_6_) predominates in the solid state, solutions in non‐coordinating solvents, and the gas phase. The dimer consists of two AlBr_4_ tetrahedral that share a common edge and exhibits four terminal and two bridging bromine atoms (Figure 1d). In Al_2_Br_6_, the Br 3d_3/2_, and Br 3d_5/2_ binding energies of terminal bromine atoms are lower than those of bridging bromine atoms. The Al 2p_1/2_ and Al 2p_3/2_ electron binding energies of Al_2_Br_6_ are shifted to lower energy levels compared to those of AlBr_3_, as a result of the different chemical environment and binding relations in these molecules.^13^ Compared with the bond dissociation energy of Al–Br (identical) in AlBr_3_, the bond dissociation energy of Al–Br (terminal) in the dimer is much closer to that of Pb–Br bond. The small disparity in bond energy and the pre‐existing Al–Br (bridging) bond of the precursor, which feature Al (III) directly bound to host lattice constituents in cluster form, favoring aluminum inclusion (as shown in Figure 1d). In our case, emission spectra of doped CsPbBr_3_ NCs shows another weak emission with a PL peak position centered at about 510 nm, which arises from the remaining undoped CsPbBr_3_ NCs. To increase the rate of successful doping, more detailed theoretical calculations for a quantitative analysis are still in research.

We rationalize the aluminum incorporation path qualitatively by invoking fundamental considerations about binding relations in AlBr_3_ and its dimer. As a means for validating this path, we attempted the doping of CsPb(Br/I)_3_ NCs with the same precursor, noting that the incorporation of aluminum atoms are in Al–Br (bridging) cluster form, suggesting that doping should introduce additional Br atoms to the lattice. Moreover, the difference of the bond dissociation energy between Pb‐I and Al–Br is larger than that between Pb–Br and Al–Br, suggesting that Al–Br (bridging) cluster should possess relatively stronger binding affinity with Br site. Indeed, as shown in Figure 1, the NCs synthesized with the same Al‐doping concentrations (≈0.2%) as that in Al:CsPbBr_3_ NCs evinced an even bigger blueshift in both absorption and emission (from 607 to 536 nm) indicative of the synergistic effect from Al‐doping and extra Br atoms incorporation. The feed ratios of Br/I in both doped and undoped CsPb(Br/I)_3_ were controlled to be 1:1. Interestingly, the Br/I ratio in the mixed halide Al:CsPb(Br/I)_3_ perovskite NCs determined according to the energy dispersive X‐ray spectroscopy (EDS) is bigger than that in pure CsPb(Br/I)_3_ NCs (Figures S4 and S5, Supporting Information), indicating the formation of mixed Al–Br/Pb–Br lattice is easier than that of Al–Br/Pb‐I one, which in turn implies the retention of at least some Al–Br (bridging) bonds during incorporation.

In principle, Al^3+^ ions in the lattice will work as donor and may induce PL quenching. However, the experimental results showed that Al doping (≈0.2%) result in a blueshift of the PL bands, without much loss of the photoluminescence quantum yields (PLQYs). As shown in Table S1 in the Supporting Information, the PLQYs of CsPb(Br/I)_3_, Al:CsPb(Br/I)_3_, CsPbBr_3_, and Al:CsPbBr_3_ NCs were 48%, 40%, 78%, and 42%, respectively. For comparison, the PL peak position of CsPb(Br/Cl)_3_ was controlled to much the same as that of Al:CsPbBr_3_ NCs (456 nm). The PLQY of Al:CsPbBr_3_ was still higher than that of CsPb(Br/Cl)_3_ (≈32%).

Colloidal CsPbX_3_ (*X* = Cl, Br, I) NCs are being explored extensively as an interesting variety of defect‐tolerant materials, wherein high efficiencies of optical and optoelectronic processes can be achieved even in the presence of defects.^14^ The defect tolerance feature in CsPbBr_3_ can be attributed to the lacking of bonding–antibonding interaction between the conduction bands and valence bands.^15^ In our case, first‐principles calculations show that partial substitution of Pb (II) by Al(III) introduces a new level in the bandgap. Considering the limited doping content obtained from experimental measurements, the Al_Pb_ defect is most probable to form a shallow state near the conduction band minimum. If the defect state lies within valence/conduction band, or forms a shallow state near the valence band maximum and the conduction band minimum, then such a defect state will be nearly delocalized in nature and will not form an efficient localized trap state.

Defect properties of a semiconductor are always critical to its performance. For example, the life time can be greatly affected by defects. Multiexponential decay for the CsPbX_3_ (*X* = I, Br, Cl) nanocrystals with lifetimes from ≈1 to ≈30 ns were reported.[[qv: 3b,16]] Multiexponential decay in CsPbX_3_ NCs should be related to their intrinsic defects, such as vacancies that have low formation energies.^17^ Time‐resolved photoluminescence (TRPL) decays of doped CsPbBr_3_ and CsPb(Br/I)_3_ nanocrystals were monitored at 456 and 536 nm, displaying triexponential decay for both Al‐doped samples (Figure 1c). Therefore, the average lifetime (τ_avg_) was calculated. For comparison, the TRPL data of undoped CsPbBr_3_ and CsPb(Br,I)_3_ were also listed in Table S2 in the Supporting Information. The average lifetime of doped CsPbBr_3_ and CsPb(Br/I)_3_ nanocrystals was calculated to be 14 and 33 ns, respectively, which reveals that τ_avg_ is of the same order of magnitude (≈10 ns) in all above cases. Furthermore, we note that the higher the PL energy, the shorter τ_avg_, as expected based on Fermi's golden rule. Abnormally, the τ_avg_ of Al doped CsPb(Br/I)_3_ is shorter than that of CsPbBr_3_. In addition to the intrinsic defects, the triexponential decay and the shorter lifetimes in Al doped CsPbBr_3_ and CsPb(Br/I)_3_ should be ascribed to the energy transfer related to the energy lever introduced by Al incorporation.

**Figure 2:**
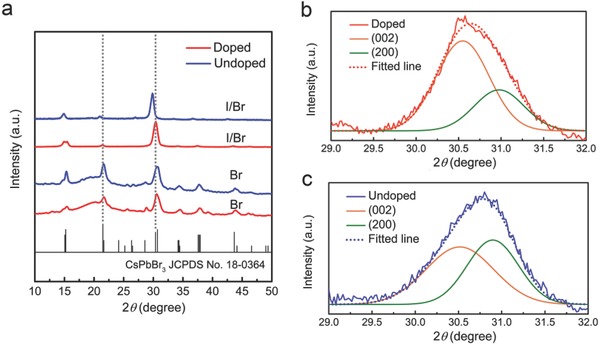
a) XRD patterns of Al‐doped and undoped CsPbBr_3_ and CsPb(Br/I)_3_ nanocrystals films. X‐ray λ = 1.54056 Å. JCPDS No.18‐0364 of the orthorhombic CsPbBr_3_ structure are indicated at the bottom of the figure. b,c) A subtle yet shift and variation of relative intensities of (200) and (002) XRD peaks is the result of lattice deformation by doping.

To obtain more detailed structural and morphological information on Al‐doped perovskite NCs, they were further examined using X‐ray diffraction (XRD) and transmission electron microscopy (TEM). As shown in **Figure** **2**a, the powder XRD data demonstrated that the Al‐doped CsPbBr_3_ and Al‐doped CsPb(Br/I)_3_ nanocrystalsare highly crystalline, composed of single orthorhombic phase, and structurally identical to their undoped host NCs. It is known that the probability of forming (or not) stable perovskite ABX_3_ (B site: divalent cation) structure or layered perovskite A_3_B_2_X_9_ (B site: trivalent cation) structures can be estimated using the Goldschmidt tolerance factor (*t*) and the octahedral factor (μ). They are based on the ionic radii (r) of the A, M, and X. According to the requirement of the octahedral factor (*μ = R*_M_/*R*_X_, 0.442 < μ < 0.895), layered perovskite A_3_B_2_X_9_ based on Al‐X octahedrons (0.243 < μ < 0.296) would not be formed. This implies that the existence form of Al would be Al_Pb_ or Al_i_, and the phase separation in the doped nanocrystals of this study is not an issue. The comparison of the diffraction angles (2θ) of several planes in undoped and doped nanocrystals are listed in **Table** **1**. Obviously, the peaks of doped CsPb(Br/I)_3_ NCs are shifted to larger angles. This is consistent with lattice contraction due to the substitution of Pb^2+^ ions (119 pm) with smaller Al^3+^ ions (53.5 pm). On the other hand, the change of Br/I ratio demonstrated above also contributes to the peak shifting. For doped CsPbBr_3_ NCs, the peak shifting seems to be less prominent. At the same time, after careful examination of the XRD patterns by peak‐differentiation‐imitating, these peaks slightly move toward to larger angles as well, as shown by the (200) and (002) peaks in Figure 2b,c. It is noted that the fitting result shows the variation of relative intensities of (200) and (002) peaks, which means the deformation of crystal particles and a preferential orientation of (002) plane (*c*‐axis).

**Table 1:** X‐ray diffraction angle (2θ) of perovskite nanocrystals

Sample [*hkl*]	CsPbBr_3_	Al‐doped CsPbBr_3_
(002)	30.49°	30.55°
(200)	30.90°	30.97°

**Figure 3:**
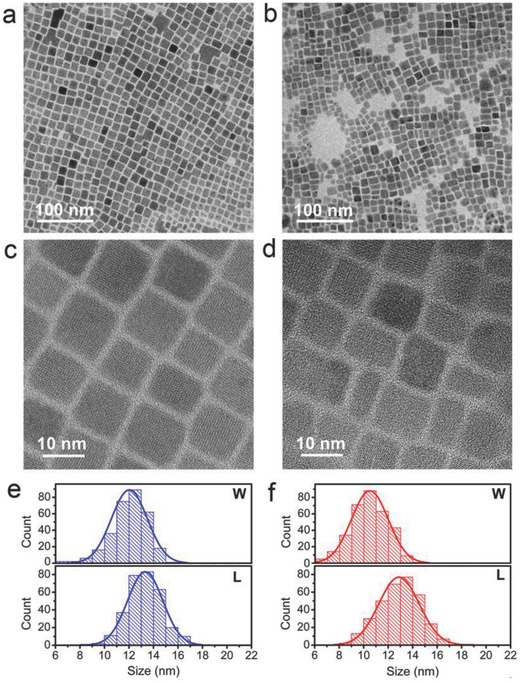
TEM images of different perovskite nanocrystals. a,c) CsPbBr_3_ and b,d) Al:CsPbBr_3_. The distributions of length and width of e) CsPbBr_3_ and f) Al:CsPbBr_3_ nanocrystals. For undoped nanocrystals, the average length is 12.02 nm and the average width is 13.33 nm. For doped nanocrystals, the average length is 10.49 nm and the average width is 12.95 nm.

TEM images of CsPbBr_3_ and Al:CsPbBr_3_ NCs were shown in **Figure** **3**, illustrating the corresponding morphological change in Al‐doped perovskite nanocrystals. The as‐prepared CsPbBr_3_ NCs presented a nearly cubic morphology; the ratio of length to width (*L*/*W* ratio) is 1.11. The doped NCs exhibited reduced size and elongated shape with the *L*/*W* ratio of 1.23. For undoped nanocrystals, the average length is 12.02 nm and the average width is 13.33 nm. For doped nanocrystals, the average length is 10.49 nm and the average width is 12.95 nm. In those quantum‐confined nanocrystals, observed spectral shifts could be caused by such size changes upon doping. Besides, asymmetric of the PL peak (tail on red‐side) at ≈456 nm may be caused by the broadening of the size distribution upon doping, as shown in Figure 3f.

The optimized crystal parameters of Al:CsPbBr_3_ listed in Table S3 in the Supporting Information shows a tendency that the c/a ratio augment with the dopant concentration, implying Al (III) ions can be accommodated as a Pb (II) substitution in host crystals with a little contraction of the lattice. The successful substitution compatibility was confirmed by experimental characterizations and theoretical calculation, either from the perspective of electronic structure or morphology changes.

**Figure 4:**
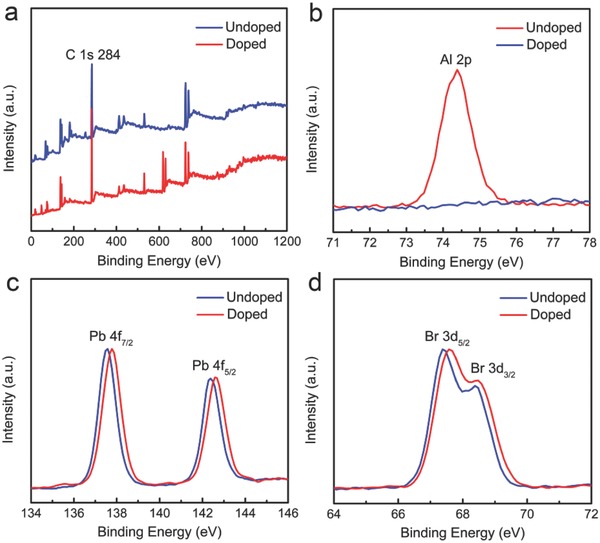
a) XPS survey spectra of CsPbBr_3_ and Al:CsPbBr_3_ NCs films. b) High‐resolution XPS spectrum of Al 2p of Al:CsPbBr_3_ NCs film. c) High‐resolution XPS spectra of Pb (4f_5/2_, 4f_7/2_) and d) Br (3d_3/2_, 3d_5/2_) of CsPbBr_3_ and Al:CsPbBr_3_ NCs films.

X‐ray photoelectron spectroscopy (XPS) analysis of the high‐resolution spectra involving Al 2p, Pb 4f, and Br 3d was then conducted to further detail the existence of Al dopant in the host NCs and their electronic states. As shown in **Figure** **4**, the Pb 4f spectrum for the pristine NCs film has been recorded with two contributions 4f_5/2_ and 4f_7/2_ locating at 142.96 and 138.10 eV, respectively, which correspond to Pb^2+^ cations. The two Br 3d signals, corresponding to 3d_3/2_ and 3d_5/2_ peaks, are also clearly seen in the Br 3d spectrum. As for the Al:CsPbBr_3_ NCs film, the Pb 4f_5/2_ and 4f_7/2_ peaks in Pb 4f spectrum as well as Br 3d_3/2_ and 3d_5/2_ peaks in Br 3d spectrum have shifted to higher binding energies, indicating the modified chemical environment of [PbBr_6_]^4−^ octahedra and the stronger Pb–Br interactions after Al incorporation. This is further evidenced by the presence of Al 2p peaks at 74.34 eV corresponding to Al (III) cations.

Distortion and tilting of the PbX_6_ octahedral is known to induce bandgap and PL changes. These distortions are always accompanied by lattice constant changes. Al is much smaller than Pb (53.5 vs 119 pm); this results in a lattice contraction of Al‐doped CsPbX_3_ NCs. The contraction of the PbBr_6_ octahedral leads to shorter Pb–Br bonds and therefore stronger interactions. This strengthens the lattice stability under the temperature variation. The thermal stability of perovskite NCs is an important factor for LED applications. To test the thermal stability, we performed PL intensity evaluation of perovskite NCs film under controlled temperature range from 20 to 100 °C. The relative PL intensity of CsPbBr_3_ NCs film decreased when the temperature increased. The thermal cycling results were shown in Figure S6 in the Supporting Information. Blue Al:CsPbBr_3_ NCs film exhibited higher thermal stability and thermal recycling than green CsPbBr_3_ NCs film. When the temperature was decreased to room temperature, the intensity of blue Al:CsPbBr_3_ NCs film was nearly the same as that before the heating treatment. The relative intensity of green CsPbBr_3_ NCs film was decreased to 60% after the heating treatment. This demonstrated that our blue Al:CsPbBr_3_ NCs offers enhanced thermal stability in terms of their emission properties, which is rather similar to the previously reported results for bulk doping perovskite‐based compounds.[[qv: 8a]]

Blue Al:CsPbBr_3_ NCs not only exhibits better stability but also avoids the color drifting issues caused by anion exchange in mixture of different halide NCs. The mixture spectra of blue CsPb(Cl/Br)_3_ and green CsPbBr_3_ with emission wavelengths of 450 and 515 nm, respectively, are illustrated in Figure S7a in the Supporting Information. The narrow emission spectra of blue and green strongly shifted to cyan, and became widened at the same time. Considering the strong anion exchange effect, we cannot use the narrow and highly efficient emission of perovskite NCs for LEDs. In this work, green CsPbBr_3_ and blue Al:CsPbBr_3_, sharing the common halogen element Br, were mixed in silicone resin and then dropped in the UV chip (365 nm). No spectrum shift was observed in the obtained spectra shown in Figure S7b in the Supporting Information. Considering the shorter Al–Br bonds and the stronger Pb–Br interactions caused by lattice contraction, the energy barrier for Br migration could be increased. It will benefit the hindering the ion migration. Even if the diffusion of halide vacancies still exists, it is a Br‐for‐Br exchange that will not change the emission spectra. So the Al:CsPbBr_3_ NCs can be used, especially in perovskite NCs mixture system, to provide the blue component of the white light for UV‐WLEDs.

**Figure 5:**
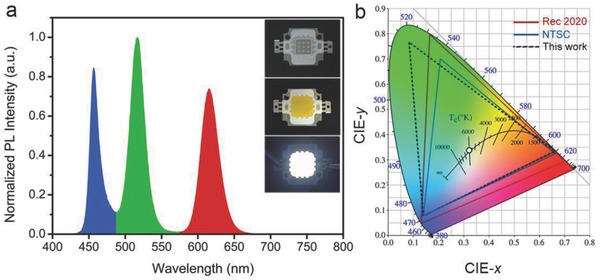
a) Emission spectrum of the WLED. The insets are the photographs of UV‐LED chips before and after encapsulation with a blend of RGB NCs. There exists no obvious agglomeration of the nanoparticles in silicone resin. The thickness of the NCs down‐conversion layer was optimized in the range 50–120 µm. b) Color gamut of the WLED in this work compared to the NTSC TV standard and the Rec. 2020 standard. The CIE color coordinates of the WLED device. For Rec 2020, the RGB CIE coordinates were (0.70, 0.29), (0.17, 0.79), and (0.13, 0.04). For NTSC, the RGB CIE coordinates were (0.67, 0.33), (0.21, 0.71), and (0.14, 0.08).

To demonstrate the ability of blue‐emitting Al:CsPbBr_3_ NCs to generate white light in combination with other luminophores, green‐emissive CsPbBr_3_ NCs and red‐emissive CdSe@ZnS NCs were used to fabricate surface mounted device type WLEDs. Emission spectrum of the WLED was shown in **Figure** **5**a. The color coordinates of the white LED were optimized at (0.32, 0.34) in CIE 1931 and a luminous efficiency of 21.6 lm W^−1^ was achieved. This is the first report of UV‐WLEDs based on doped perovskite NCs. For backlight display, the NTSC value of the device should be calculated. The color filter was used to collect the red, green, and blue colors from the UV pumped perovskite NCs‐white LED. Before the NCs were filtered, the emission wavelengths of red‐green‐blue (RGB) were 615, 515, and 456 nm. After they passed through the color filter, the RGB CIE coordinates were (0.66, 0.34), (0.08, 0.77), and (0.14, 0.08). Coincidently, the location of blue color generated from Al:CsPbBr_3_ perovskite NCs and that in NTSC standard were overlap. The color gamut overlap of the NTSC space was 116%, which was higher than a previously designed regular phosphor LED (NTSC 86%) and Cd‐QD LED (NTSC104%). This result is attributed to the narrow emission wavelength of the green CsPbBr_3_ and blue Al:CsPbBr_3_ perovskite NCs. Rec. 2020 color space (UHDTV) is a newly released ITU‐R Recommendation, CIE 1931 chromaticity diagram in Figure 5b showing the color space in the triangle and the location of the primary colors. For a wider color gamut, the new definition of the RGB CIE coordinates were (0.70, 0.29), (0.17, 0.79), and (0.13, 0.04). The overlap area of the Rec. 2020 of the UV pumped perovskite NCs‐white LED was 87%.

Besides 2D perovskites nanoplatelets and hybrid composite films with blue photoluminescence (see Table S4, Supporting Information),^18^ our work introduces a new member to blue‐emissive lower dimensional perovskites. When this manuscript was ready for submission, we noticed a new publication on the same topic. Divalent ion (Sn^2+^, Cd^2+^, Zn^2+^) doped CsPbBr_3_ NCs exhibit blueshift PL, but only Cd‐doped CsPbBr_3_ NCs can reach the region of desired deep‐blue.[[qv: 16a]] Comparing to their results, we are the first to show doping with a trivalent metal ion causing a blueshift of the PL emission, and the Al:CsPbBr_3_ NCs are more eco‐friendly.

## 3 Conclusion

In summary, we have demonstrated a practical doping strategy that can be applied to lead‐halide perovskites NCs, leading to the realization of stable blue photoluminescence by inclusion of appropriate Al (III) precursors (AlBr_3_) during NC growth. The small disparity in bond strengths within the precursor and the host lattice, and the pre‐existing Al–Br (bridging) bond, which feature Al directly bound to host lattice constituents in cluster form, favoring Al inclusion. In this way, cation compositional engineering of perovskite NCs can be employed as an additional way to rationally tune the photophysical properties, making them luminesce in a desired spectral range. Further, partial lead cation substitution without altering the halide composition provides a feasible approach to avoid fast anion‐exchange effect. Al:CsPbBr_3_ NCs based UV‐pumped WLEDs exhibit an NTSC value of 116% and Rec. 2020 of 87%. We anticipate that our present work will stimulate further research to exploit foreign‐cation‐doped lead halide perovskites NCs and make all perovskites RGB NCs based optoelectronic devices with no obstruction of anion‐exchange effect.

## 4 Experimental Section

*Materials and Chemicals*: Cesium carbonate (Cs_2_CO_3_, Alfa Aesar, 99.9%, metals basis), lead (II) bromide (PbBr_2_, Alfa Aesar, 99.999%, metals basis), lead (II) iodide (PbI_2_, Alfa Aesar, 99.999%, metals basis), aluminum bromide (AlBr_3_, Alfa Aesar, 99.997%, metals basis), oleylamine (OAm, Aldrich, technical grade 70%), oleic acid (OA, Aldrich, technical grade 90%), 1‐octadecene (ODE, Aldrich, technical grade 90%).

*Preparation of Cs‐Oleate*: Following the recipe reported by Kovalenko and co‐workers. Cs_2_CO_3_ (0.640 g), dried OA (2 mL), and dried ODE (24 mL) were added to a 50 mL 3‐neck round bottomed flask and then heated to 150 °C until all Cs_2_CO_3_ reacted with OA. It was preheated to 140 °C just before injection. All the reactions were conducted in a nitrogen‐filled glovebox (0.1 ppm H_2_O; 0.1 ppm O_2_).

*Synthesis of CsPbX_3_ NCs (X = Br, Br/I, Cl/Br)*: Dried ODE (5 mL) and PbX_2_ (0.188 mmol) such as PbBr_2_ (0.069 g) or their mixtures PbBr_2_/PbI_2_ (0.035 g/0.043 g, molar ratio 1:1), PbCl_2_/PbBr_2_ (0.026 g/0.035 g, molar ratio 1:1), were loaded into 25 mL 3‐neck flask. Dried OAm (0.5 mL) and OA (0.5 mL) were then injected at 120 °C. After complete solubilization of a PbX_2_ salt, the temperature was raised to 150 °C and Cs‐oleate solution (0.4 mL) was quickly injected. 10 seconds later, the solution was transferred to an included icebox in the glove box for cooling.

*Synthesis of Al‐Doped CsPbX_3_ NCs (X = Br, Br/I)*: Dried ODE (5 mL), AlBr_3_ (0.030 g), PbX_2_ such as PbBr_2_ (0.069 g) or their mixtures PbBr_2_/PbI_2_ (0.035 g/0.043 g, molar ratio 1:1), dried OAm (0.5 mL) and OA (0.5 mL). Then, the temperature was raised to 150 °C. Dried OAm (0.3 mL) and dried OA (0.3 mL) were subsequently injected to solubilize the solution. Then Cs‐oleate solution (0.4 mL) was quickly injected. 1 min later, the solution was transferred to an included icebox in the glove box for cooling.

*Isolation and Purification of Cs‐Based NCs*: The NCs were precipitated out of ODE at room temperature and separated using centrifugation and decanting of the supernatant. The solid NC product was then dispersed in toluene. For additional washing, the NCs were precipitated with acetonitrile and centrifuged followed by dissolving in toluene.

*Fabrication of WLEDs*: A certain amount of blue, green, and red NCs (mass ratio 4:4:1) were mixed with 0.1 g of thermal‐curable silicone resin OE‐6551A under vigorous stirring. Subsequently, the hardener (OE‐6551B, 0.2 g) was added to form a paste. After bubbles were removed, the paste was dropped onto a GaN‐based UV‐LED and then cured.

*Characterization*: UV–vis absorption spectra for colloidal solutions were collected with a PerkinElmer Lambda 950 spectrophotometer on dilute toluene solutions in a 1 cm × 1 cm quartz cuvette. The PL spectra were obtained by a PerkinElmer LS 55 fluorescence spectrometer. PL lifetimes were recorded on Hamamatsu compact fluorescence lifetime spectrometer. Lifetime data were analyzed with U11487 software package. Absolute emission quantum yields were recorded on Hamamatsu absolute PL quantum yield spectrometer C11347 equipped with an integrating sphere. Inductively coupled plasma mass spectrometry (HORIBA Jobin Yvon JY2000‐2) was used to determine the amount of Cs, Pb, and Al present in undoped and Al‐doped CsPbBr_3_ and CsPb(Br/I)_3_ perovskite nanocrystals. The nanocrystal solutions were sonicated in concentrated nitric acid to ensure complete digestion of the sample. The ratio between the bromide and iodide in the mixed halide perovskite was determined using SEM/EDS (ZeissSupra 55) operated at 5 kV. XRD patterns were collected using Bruker D8 Advance diffractometer with a Cu Kα (0.15418 nm) X‐ray source, operating at 40 kV and 40 mA. To prepare the sample, a concentrated NC solution in toluene was drop‐cast onto a previously cleaned square silicon wafer. Conventional TEM observations were carried out using a JEOL JEM 3200FS microscope equipped with a thermionic gun operating at 300 kV of accelerating voltage. Samples were prepared by dropping diluted nanocrystal solutions onto carbon‐coated 200 mesh copper grids with subsequent solvent evaporation. XPS measurements were carried out in a Thermo Fisher Escalab 250Xi ultrahigh vacuum surface analysis system. For the thermal stability test, the heating ramp was 10 °C min^−1^, the temperature of the sample was monitored by infrared detection gun (±0.1 °C). The emission spectra and the photometric characteristics of UV‐pumped WLEDs were measured on a high accuracy array rapid spectroradiometer (SpectraScan PR‐705 with a MS‐55 lens).

## Conflict of Interest

The authors declare no conflict of interest.

## Supporting information

Supplementary
