# Supplementary material for: Aluminum‐Doped Cesium Lead Bromide Perovskite Nanocrystals with Stable Blue Photoluminescence Used for Display Backlight
Source: Adv Sci (Weinh). 2017 Jul 31;4(11):1700335. doi: 10.1002/advs.201700335 (PMC5700652; doi:10.1002/advs.201700335)
Supplement: Supplementary file 1 — Supplementary [file ADVS-4-na-s001.pdf]

Copyright WILEY-VCH Verlag GmbH & Co. KGaA, 69469 Weinheim, Germany, 2017.

ADVANCED SCIENCE Open Access

Supporting Information

for *Adv. Sci.,* DOI: 10.1002/advs.201700335

Aluminum-Doped Cesium Lead Bromide Perovskite Nanocrystals with Stable Blue Photoluminescence Used for Display Backlight

*Ming Liu, Guohua Zhong, Yongming Yin, Jingsheng Miao, Ke Li, Chengqun Wang, Xiuru Xu, Clifton Shen, and Hong Meng**

Supporting Information

Aluminum-Doped Cesium Lead Bromide Perovskite Nanocrystals with Stable Blue Photoluminescence Used for Display Backlight

*Ming Liu, Guohua Zhong, Yongming Yin, Jingsheng Miao, Ke Li, Chengqun Wang, Xiuru Xu, Clifton Shen and Hong Meng **

Computational Methods

Total energy and electronic structures calculations for polymorphs of $CsPb_xAl_{1-x}Br_3$. In the first-principles calculation, we employed the Vienna Ab-initio Simulation Package (VASP)[1] based on the projector augmented wave (PAW) [2] method with a cutoff energy of 300 eV. All of configurations of $CsPb_xAl_{1-x}Br_3$ were fully optimized using a conjugate-gradient algorithm. The Monkhorst-Pack *k*-point grids are generated according to the specified *k*-point separation of 0.02 $Å^{-1}$ and the convergence thresholds are set as 10^{-6} eV in energy and 10^{-3} eV/Å in force. The generalized gradient form (GGA) of the exchange-correlation functional (Perdew-Burke-Ernzerhof96, PBE) was adopted.[3] And considering the non-local interaction, we has added the correction of van der Waals (vdW) in version of vdW-DF2 in this calculation.[4] The band structures were calculated along high symmetry k-point paths to investigate the change of energy band characteristics induced by doping, and the density of states were simulated to analyze the electronic states near Fermi level and the contribution of impurity.

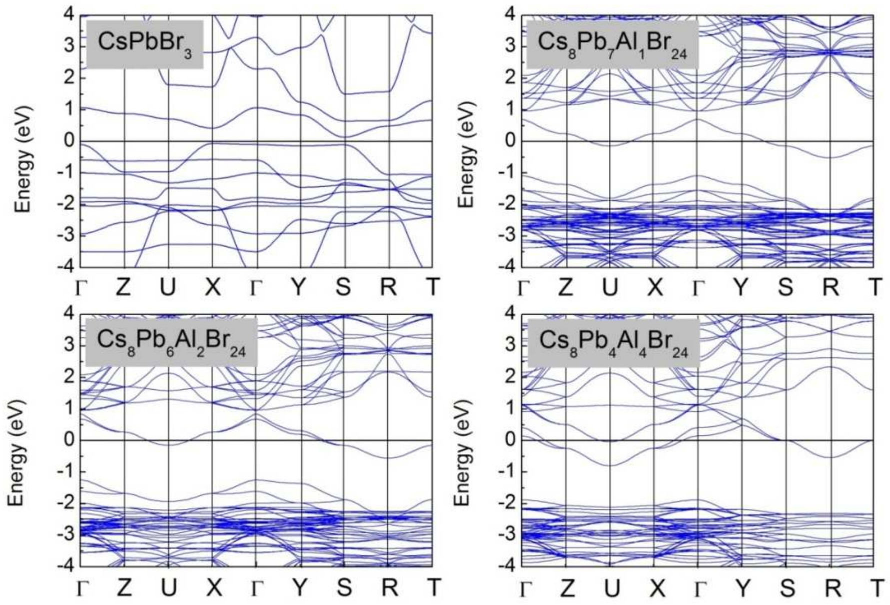

Figure S1. The calculated electronic band structures for the Al-doped cesium lead bromide perovskites (cubic phase) with different Pb-Al ratio.

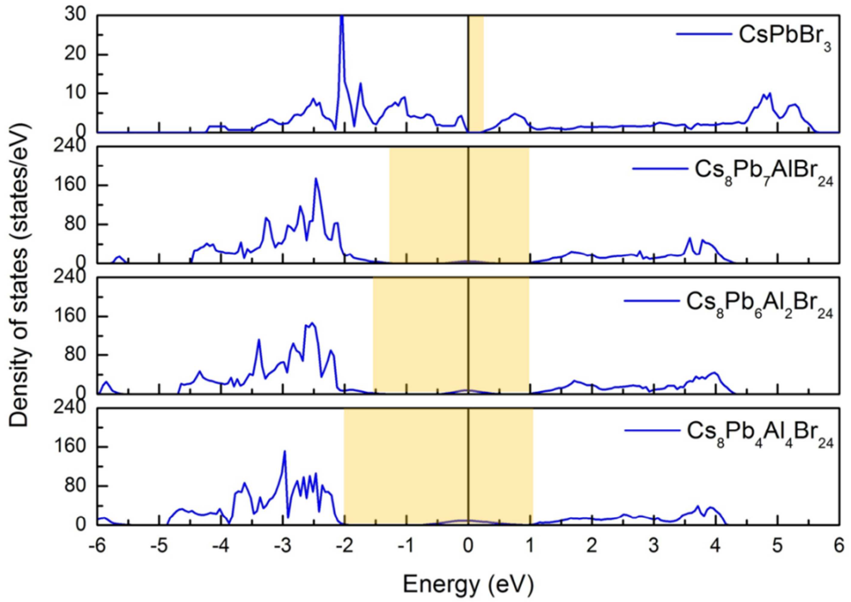

Figure S2. The calculated density of states for the Al-doped cesium lead bromide perovskites (cubic phase) with different Pb-Al ratio. Visualized band gap was shown in the shaded parts.

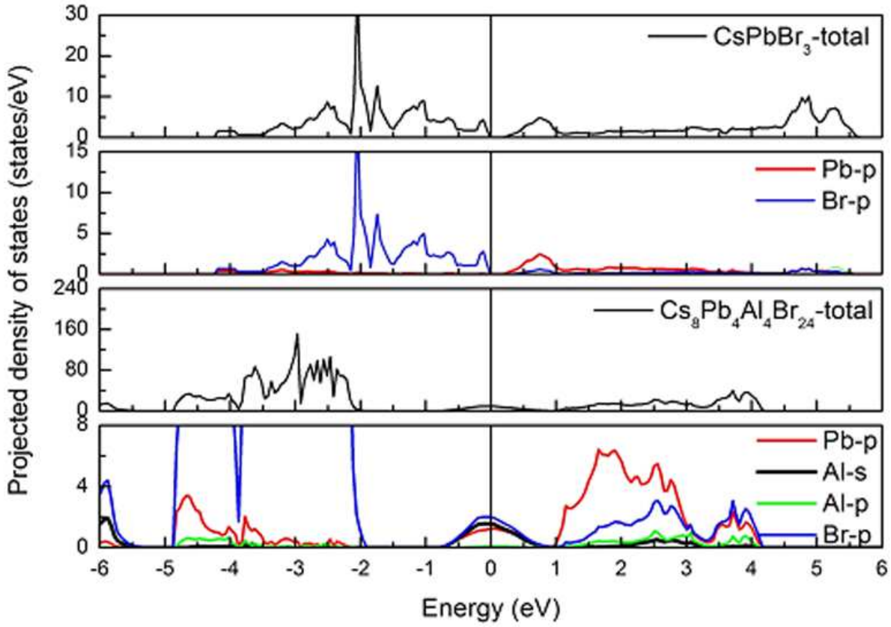

Figure S3. The calculated prejecteddensity of states for the Al-doped cesium lead bromide perovskites (cubic phase) with Pb-Al ratio of 1.

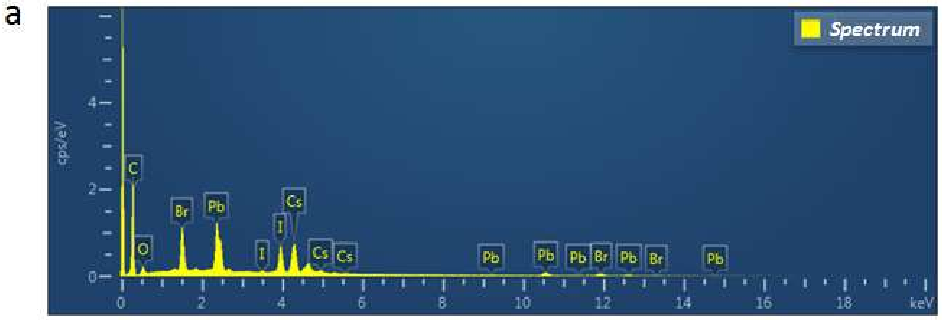

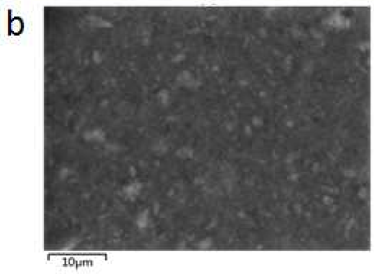

c

Element	*Atom %*
C	90.36
O	3.08
Br	1.75
I	1.81
Cs	1.54
Pb	1.46
Total	100.00

Figure S4. SEM/EDS atomic composition of $CsPb(Br/I)_3$ perovskite nanocrystals.

a

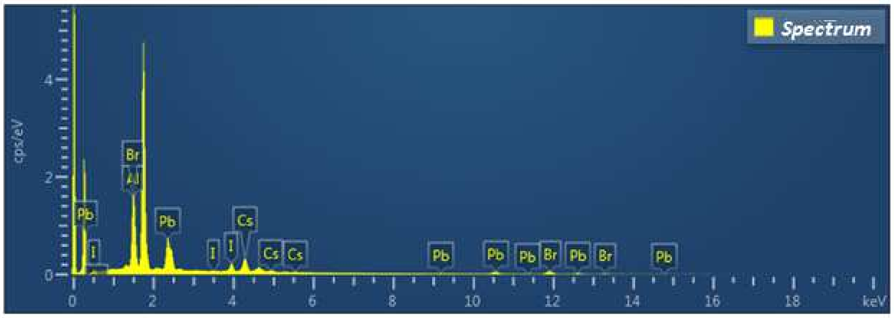

b

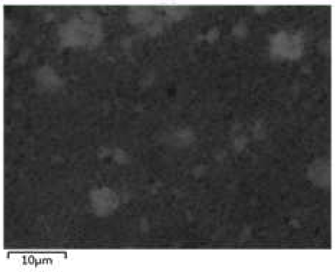

c

Element	*Atom %*
C	86.12
O	2.88
Br	7.17
I	1.65
Cs	0.86
Pb	1.32
Total	100.00

Figure S5. SEM/EDS atomic composition of Al-doped $CsPb(Br/I)_3$ perovskite nanocrystals.

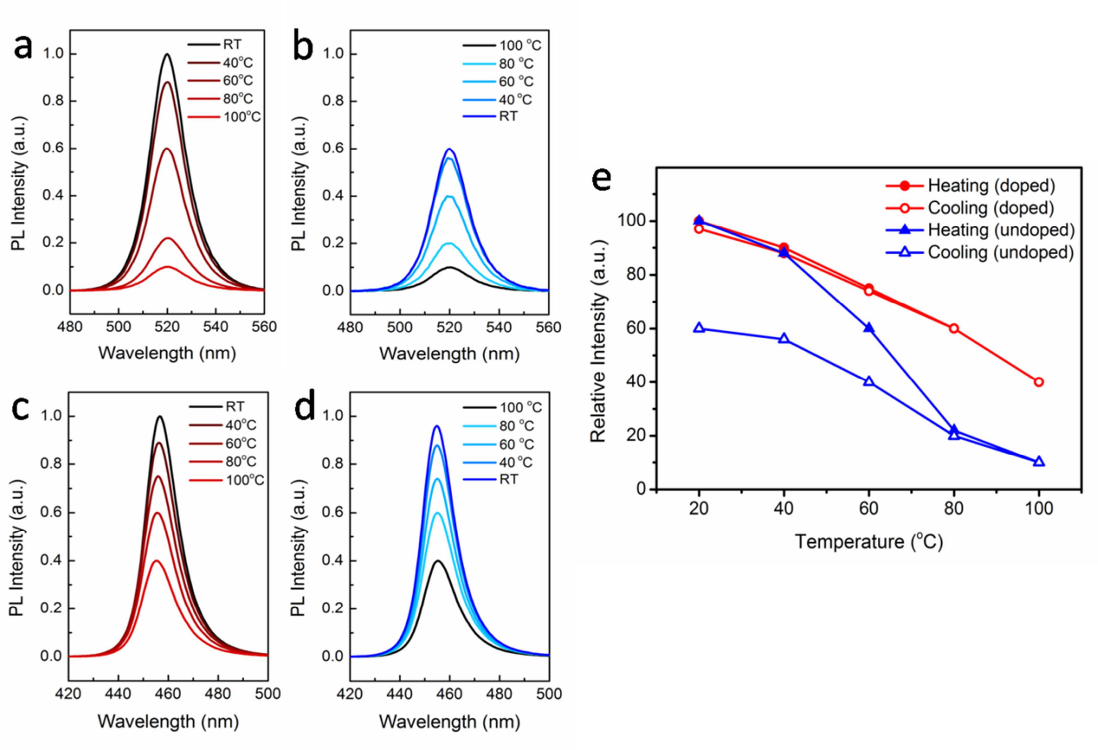

Figure S6. Thermal cycling of $CsPbBr_3$ and Al:$CsPbBr_3$. The data points in Figure e were determined according to the PL peak intensity of (a, b) $CsPbBr_3$ and (c, d) Al:$CsPbBr_3$ NCs at different temperature in the thermal cycling.

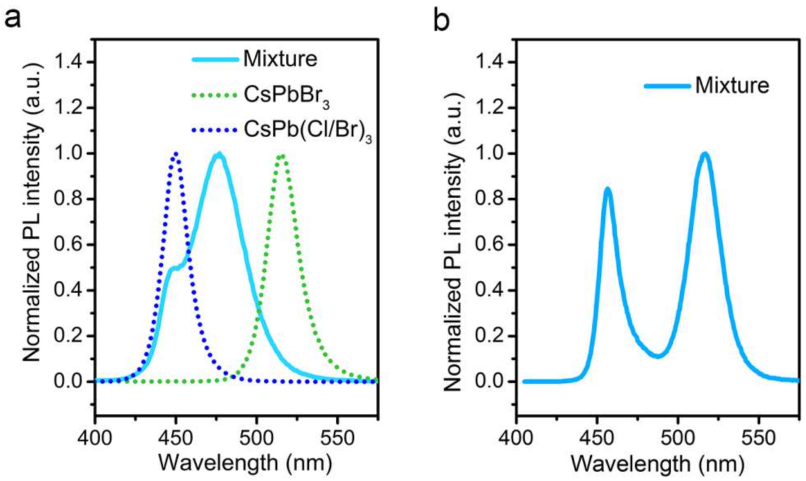

Figure S7. (a) The spectra of blue $CsPb(Cl/Br)_3$, green $CsPbBr_3$ perovskite nanocrystals, and their mixture under UV chip (365 nm) excitation. (b) The spectra of the mixture of blue Al:$CsPbBr_3$ and green $CsPbBr_3$ perovskite nanocrystals under UV chip (365 nm) excitation.

Table S1. Absolute photoluminescence quantum yields (PLQY) of the five samples

**Samples**	**PLQY(%)**
$CsPb(Br/I)_3$	48
Al-doped$CsPb(Br/I)_3$	40
$CsPbBr_3$	78
Al-doped $CsPbBr_3$	42
$CsPb(Cl/Br)_3$	32

Table S2. Fitted parameters of the decay curve for $CsPb(Br/I)_3$, Al-doped $CsPb(Br/I)_3$, $CsPbBr_3$ and Al-doped $CsPbBr_3$NCs.

**Samples**	**A_1 (%)**	**τ_1 (ns)**	**A_2 (%)**	**τ_2 (ns)**	**A_3 (%)**	**τ_3 (ns)**	**τ_{ave} (ns)**
**$CsPb(Br/I)_3$**	64.94	17.09	32.01	67.71	3.05	251.63	88.70
**Al-doped$CsPb(Br/I)_3$**	89.39	10.52	10.18	39.97	0.43	226.20	33.30
**$CsPbBr_3$**	82.25	4.68	16.57	22.93	1.18	140.52	36.89
**Al-doped $CsPbBr_3$**	97.03	5.11	2.82	34.82	0.15	139.70	14.44

Table S3. The optimized crystal parameters of Al:$CsPbBr_3$ ($\alpha=\beta=\gamma=90°$)

**Crystal parameters (Å)**	**$Cs_8Pb_8Br_{24}$**	**$Cs_8Pb_7Al_1Br_{24}$**	**$Cs_8Pb_6Al_2Br_{24}$**	**$Cs_8Pb_4Al_4Br_{24}$**
a	12.7669000626	12.0299412089161706<br>(-5.77%)	11.8849822094491770<br>(-6.91%)	11.5070029689443132<br>(-9.87%)
b	12.7669000626	12.0299412089161706<br>(-5.77%)	11.8849822094491770<br>(-6.91%)	11.5070029689443132<br>(-9.87%)
c	12.7669000626	12.0299412089161706<br>(-5.77%)	11.8963110167836792<br>(-6.82%)	11.8916024480072000<br>(-6.86%)
c/a	1	1	1.0009532	1.0334231

Table S4. Two dimensional perovskites nanoplatelets and hybrid composite films with blue photoluminescence

**Materials**	**PL peakposition**	**FWHM [nm]**	**Quantum yield[%]**	**PL lifetime [ns]**	**Ref.**
$(R_1NH_3)_2[(R_2NH_3)_2PbBr_4]_{(n-1)}PbBr_4$	413	11	16	3.12	*Chem.Commun.* ***2015****, 51,16385*
$(C_4H_9NH_3)_2PbBr_4$	406	–	26	3.3	*Science* ***2015****,349, 1518*
$CsPbBr_3$	488	14	84.4	–	*J. Am. Chem. Soc.* ***2015****, 137, 16008*
$CsPbBr_3$	459	–	31	3	*J. Am. Chem. Soc.* ***2016****, 138, 1010*
$CsPbBr_3$	458	13	33	4.3	*J. Am. Chem. Soc.* ***2016****, 138, 7240*
$CsPbCl_3$	412	8	–	–	*Adv.Funct.Mater.* ***2016****, 26, 6238*
This work Al:$CsPbBr_3$	456	16	42	14	

References:

(1) Kresse, G; Joubert D. *Phys Rev B* 1999, 59, 1758.

(2) Ceperley, D. M.; Alder, B. J. *Phys Rev Lett* 1980, 45, 566.

(3) Perdew, J. P.; Burke, K.; Ernzerhof, M. *Phys Rev Lett* 1996, 77, 3865.

(4) Lee, K.; Murray, É. D.; Kong, L.; Lundqvist, B. I.; Langreth, D. C. *Phys Rev B* 2010, 82, 081101.
